# Acceptability and Satisfaction of Patients and Providers With Telemedicine During the COVID-19 Pandemic: A Systematic Review

**DOI:** 10.7759/cureus.56308

**Published:** 2024-03-17

**Authors:** Shazina Saeed, Manmohan Singhal, Karuna N Kaur, Mohd. Shannawaz, Arunima Koul, Kanika Arora, Bhavna Kumar, Neeraj Kumar Sethiya, Shamimul Hasan

**Affiliations:** 1 Amity Institute of Public Health & Hospital Administration, Amity University, Noida, IND; 2 School of Pharmaceuticals and Population Health Informatics, DIT University, Dehradun, IND; 3 Oral Medicine and Radiology, Faculty of Dentistry, Jamia Millia Islamia, New Delhi, IND

**Keywords:** covid-19 pandemic, telemedicine (tm), provider satisfaction, patient’s satisfaction, covid-19

## Abstract

COVID-19, also known as coronavirus disease 2019, is an extremely contagious viral sickness caused by severe acute respiratory syndrome coronavirus 2 (SARS-CoV-2). After the first cases of this primarily respiratory viral illness were recorded in Wuhan, Hubei Province, China, in late December 2019, SARS-CoV-2 rapidly disseminated across the globe. Consequently, on March 11, 2020, the World Health Organization (WHO) declared it a global pandemic. The rapid spread of the COVID-19 virus, coupled with subsequent lockdowns and social distancing measures, profoundly disrupted traditional healthcare delivery systems. Amidst the COVID-19 pandemic, telemedicine emerged as a pivotal solution for delivering healthcare services while minimizing exposure to the virus. This study aims to assess patient and provider satisfaction with telemedicine during this unprecedented period. A systematic literature search was conducted on PubMed and Google Scholar using specific MeSH terms and Preferred Reporting Items for Systematic Literature Reviews and Meta-Analyses (PRISMA) guidelines to summarize patient and provider satisfaction concerning telemedicine using all the facts, evidence, and published literature. The analysis showed that although providers were generally satisfied with telemedicine, they were less satisfied than patients due to technical issues and difficulties transmitting documents. Patients reported high satisfaction with telemedicine, citing convenience and cost savings as major benefits. However, a lack of provider compensation was identified as a potential barrier to adoption. Most providers believed that telemedicine was only necessary in emergencies while a few recognized its potential for routine care. The study concludes that telemedicine has the potential to improve healthcare access and efficiency, but more research is needed to address technical and reimbursement issues and to determine the appropriate scope of telemedicine use. Overall, the findings of this study can inform future healthcare policies and regulations to ensure that telemedicine is used effectively and to the satisfaction of both patients and providers.

## Introduction and background

COVID-19, also known as coronavirus disease 2019, is caused by the severe acute respiratory syndrome coronavirus 2 (SARS-CoV-2) infection. COVID-19 displays a diverse range of symptoms, with the majority of cases being mild infections (80%). Nevertheless, 20% of those infected may experience severe illness, and 5% may progress to critical conditions, leading to pneumonia or acute respiratory distress syndrome, thus requiring mechanical ventilation and hospitalization in intensive care units [1].

COVID-19 patients with underlying comorbidities, such as hypertension, diabetes mellitus, cardiac or renal disorders, etc., are at an increased risk of developing severe complications like septic shock, acute respiratory distress syndrome, and, ultimately, death [2]. The emergence of the COVID-19 virus in December 2019 rapidly escalated into a global pandemic, ultimately prompting the World Health Organization (WHO) to declare a global health emergency on March 11, 2020 [3,4].

The rapid spread of the virus and subsequent lockdowns and social distancing measures significantly disrupted traditional healthcare delivery models. In response, telemedicine emerged as a vital tool for ensuring continuity of care and minimizing the risk of virus transmission [5,6]. The COVID-19 pandemic presented an unprecedented challenge to healthcare delivery, necessitating rapid innovations and adaptations [4]. While the initial surge of telemedicine use was driven by the immediate need to respond to the pandemic, its potential to transform healthcare delivery has become increasingly evident.

According to the WHO, telemedicine is defined as “the delivery of health care services by all health care professionals using technology for the exchange of valid information for the diagnosis, treatment, and prevention of disease and injuries” [7]. Telemedicine offers numerous advantages, including increased accessibility, convenience, flexibility, and cost-effectiveness. It can also improve patient engagement and empower individuals to take a more active role in managing their health [8]. The burgeoning field of telemedicine holds immense promise for revolutionizing healthcare delivery by enhancing accessibility, convenience, and flexibility. However, the long-term implications, particularly regarding patient and provider satisfaction, remain inadequately explored. While there is existing literature on patient or provider satisfaction individually, there is a noticeable gap in research exploring their combined perspectives and acceptance of telemedicine in the aftermath of the pandemic [9].

To address this research gap, our systematic review undertakes a thorough analysis of existing literature on both patients’ and providers’ acceptability and satisfaction with telemedicine, and therefore determine whether telemedicine was a positive or negative adaptation in response to the COVID-19 pandemic. By synthesizing their perspectives, this review aims to provide a comprehensive understanding of the advantages and limitations of telemedicine. The insights gained from this review can contribute to the development of future telemedicine interventions and enhance healthcare delivery strategies.

## Review

Materials and methods

Search Strategy

This systematic review of literature utilized the 2020 Preferred Reporting Items for Systematic Literature Reviews and Meta-Analyses (PRISMA) guidelines and was registered with the Prospective Register of Systematic Reviews (PROSPERO) [10]. The registration ID of the review is CRD42023403646 and can be accessed at https://www.crd.york.ac.uk/prospero/#myprospero. 

The search for the systematic review was taken up by defining the keywords related to the population, intervention, control, and outcomes (PICO) format: (a) Population - “Patient’s and health care professionals (providers) using telemedicine as a health care delivery system during COVID-19”; (b) intervention/exposure - “telemedicine”; (c) control - “ Patient’s and health care professionals (providers) using interventions other than telemedicine and (d) outcome - “acceptability and satisfaction with telemedicine”.

Our focused research question was “to evaluate the patient’s and provider’s acceptability and satisfaction with telemedicine during COVID-19”?

The PRISMA statement comprises a checklist of 27 items that guarantee transparency, iteration, and comprehensive reporting in systematic reviews. The literature search was carried out in November 2023, using “PUBMED” and “Google Scholar” databases, and the following term sequences were combined: “patient satisfaction”, “provider satisfaction”, “healthcare workers”, “telemedicine”, and “COVID-19”. Additionally, we examined the references of these selected articles to uncover any further studies or reports that were not found during the initial searches. The following Inclusion and exclusion criteria were considered (Table 1).

**Table 1:** Inclusion and exclusion criteria

Inclusion Criteria	Exclusion Criteria
Studies focusing on both patient and provider satisfaction regarding telemedicine, original articles including both cross-sectional and mixed-method studies, full-text online access published articles, articles published between December 2019 and November 2023, and studies published and translated in the English language were included.	Articles focusing on subspecialists, i.e. oncology, orthopedics, etc.; review articles, case reports, case series, and randomized controlled trials, and articles that did not include COVID-19 patients were excluded.

Study Selection

**Figure 1:**
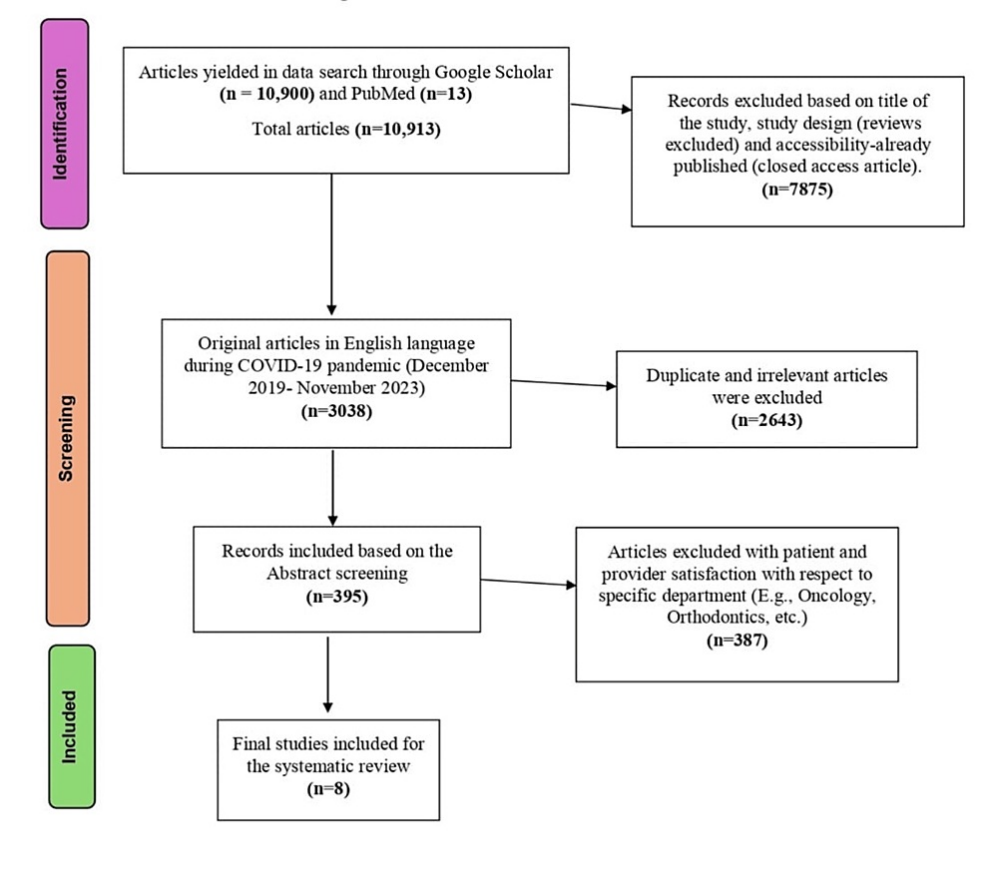
PRISMA flowchart PRISMA: Preferred Reporting Items for Systematic Literature Reviews and Meta-Analyses

To select the relevant studies on patient and provider satisfaction with telemedicine during the COVID-19 pandemic, a two-step process was used. First, the titles and abstracts of 10,913 articles published in the last two years were reviewed, and potential eligibility was assessed. Second, the full texts of these articles were obtained for a more detailed evaluation. Only articles reporting the level of satisfaction for both patients and providers for telemedicine during COVID-19 were considered for inclusion. After the initial search, 7875 articles were eliminated, as those did not fulfill the inclusion criteria, and 2,643 articles were removed due to duplication of the studies. Moreover, 387 articles that were either not relevant to patient and provider satisfaction, or telemedicine, or articles focused on specific subspecialties, such as oncology, orthodontics, dermatology, etc. were also excluded, as they may exhibit bias, potentially leading to higher satisfaction levels for both patients and healthcare providers. Finally, eight articles that met the inclusion criteria were included in the study, as illustrated in Figure 1.

Data Extraction

The information from each study was collected and organized into Microsoft Excel spreadsheets, with the following data domains extracted: study design, participant information, sample size, sampling techniques, scales used, country/state/city, outcome measures, and findings.

Risk-of-Bias Assessment

The risk of publication bias was assessed by using an R package and the Shiny web app for visualizing risk-of-bias assessments introduced by the National Institute for Health Research (NIHR), as a part of the Doctoral Research Fellowship (DRF-2018-11-ST2-048) at the University of Bristol, UK. The current version from 2020 was used for the analysis [11]. The program evaluates the following six domains: 1. randomization procedure, 2. recommended intervention, 3. missing outcome data, 4. assessment of the outcome, 5. selection of the outcome report, and 6. overall evaluation.

Results

Our initial search identified 10,913 results, and after filtering titles, study designs, and abstracts, and removing duplicates, only 8 studies met our inclusion criteria. Notably, all the studies were cross-sectional and conducted outside India (Table 2) [12-19].

**Table 2:** Descriptive characteristics of the included studies

S.no.	Author/year/country	Study design	Mode of data collection	Sampling technique	Scale used	Sample size	Age	Outcomes	Patient Satisfaction	Provider Satisfaction
1.	F. M. V. Erkel et al. 2022 Netherlands. [19]	Cross-sectional study	Interviews	Purposive sampling	Not used	82 patients and 58 healthcare professionals were interviewed.	59.1 years (patient mean age) 43.1 years (Physician mean age)	Predominantly, patients and Healthcare Professionals (HCP) were satisfied with Telemedicine. Patients believed that if there was a strong patient-HCP relationship, Telemedicine was effective, easily available, and acceptable	Patient satisfaction levels were explored through qualitative interviews	Healthcare providers (HCPs) faced challenges and expressed lower job satisfaction with telephone consultations.
2.	M. Zhao et al. 2022 Canada [15]	Mixed methods study	Surveys and virtual interviews	Convenience sampling	5-point Likert scale	85 patients and 94 Primary Care Providers	Not mentioned	Patients were more satisfied with Video Consultation than Physicians (p value< 0.001). Physicians were more satisfied than nurse practitioners and pharmacists.	Median of 4.3 with an IQR of 4.0-4.7	Median of 4.0 with an IQR of 3.3-4.3
3.	J. Volcy et al. 2021 USA [14]	Cross-sectional study	Survey Method	Convenience sampling	5-point- Likert scale	223 patients and 72 providers from Internal and family medicine	Not mentioned	Tele-visits were preferred by 84.4% of Internal Medicine consumers and 94% of Family Medicine individuals. 91% of Internal Medicine clinicians and 88% of Family Medicine physicians felt at ease managing virtual visits.	a) 84.4% of general surgery patients preferred telemedicine. 94% of family medicine patients preferred telemedicine.	a) 91% of Internal Medicine (IM) clinicians and 88% of Family Medicine (FM) clinicians felt comfortable conducting virtual sessions. 82.9% of IM doctors (47 interviewed) and 64% of FM clinicians were satisfied with video consultation (VC)
4.	H. Y. Park et al. 2021 Korea [13]	Cross-sectional study	Survey Method	Convenience Sampling	Not used	906 patients, 182 doctors, and 138 nurses.	40 (mean age of patients)	86% of patients, 52% of doctors, and 48% of nurses were satisfied with telemedicine.	a) 86% of respondents were satisfied with telemedicine. b) 87.1% rated the quality of patient-clinician contact as effective.	a) 52.7% of physicians and 48% of nurses were satisfied with the use of telemedicine, b) 7.3% of physicians and 9% of nurses were satisfied with the effective patient-medical personnel interaction.
5.	J. Yu et al. 2021 USA [15]	Cross-sectional study	Survey method	Convenience sampling	5-point Likert scale for patients	50 patients and 45 physicians	Not mentioned	84% of patients were extremely satisfied with telemedicine while 58% of physicians were not.	a) 84% were satisfied with their telemedicine experience following COVID-19. b) 72% of patients reported an interest in continued telemedicine sessions.	a) 58% of doctors were dissatisfied with telemedicine, and nearly half were worried that social networking tools would compromise the doctor-patient relationship. b) 29% of doctors thought that their patients' problems were appropriately addressed through telemedicine.
6.	Y. Wang et al. 2021 China [18]	Survey -cross-sectional study	Survey method	Convenience sampling	Not used	81 doctors and 61 patients participated in the satisfaction survey	Not mentioned	91.80% of the surveyed doctors and 68.42% of the patients were highly satisfied with telemedicine.	a) 68.42% were satisfied with the outcomes of video consultation, and b) 92.11% would recommend it further.	a) 91.80% of the doctors were satisfied with the use of telecommunication.
7.	G. Barkai et al. 2020 Israel [12]	Cross-sectional study	Survey method	Convenience sampling	10-point and 5-point Likert scales used	540 consumers, 162 physicians who used telemedicine, and 50 physicians who did not	Not mentioned	485 individuals (89.8%) were highly satisfied with telemedicine. 61 physicians (37.7%) expressed strong satisfaction with telemedicine.	a) 89.8% were satisfied with the physician and waiting period for video consultations, and b) 6.3% were moderately satisfied. c) 3.9% were dissatisfied.	Less than 40% of clinicians reported high levels of satisfaction with video consultations (VC).
8.	D. G. Huidobro et al. 2020 Chile [16]	Mixed-method survey study	Interviews for physicians and questionnaires for patients.	Convenience sampling	Not used	3962 patients and 263 physicians participated in the study.	Not mentioned	Telemedicine was rated as satisfactory by 244 providers (92.8%). Patients were less satisfied with the payment procedure and the use of the web portal.	Satisfaction was very high with both telemedicine and in-person services at a score of 96.5 and 97.4, respectively.	a) 92.8% of providers were highly impressed using telemedicine, and the majority recommend it to family and friends (94.2%).

The studies originated from diverse geographical locations, including the United States (n=2), Canada (n=1), Korea (n=1), Israel (n=1), China (n=1), Chile (n=1), and the Netherlands (n=1).

The included studies employed various methods to assess satisfaction. Four studies used numerical rating scales (5- or 10-point Likert scales) while others included dedicated questions within satisfaction questionnaires or semi-structured interviews for deeper qualitative exploration. These studies investigated diverse factors influencing patient and provider satisfaction with telemedicine, including technology utilization, user convenience, overall patient experience, system reliability, satisfaction levels, and adherence to scheduled appointments.

**Figure 2:**
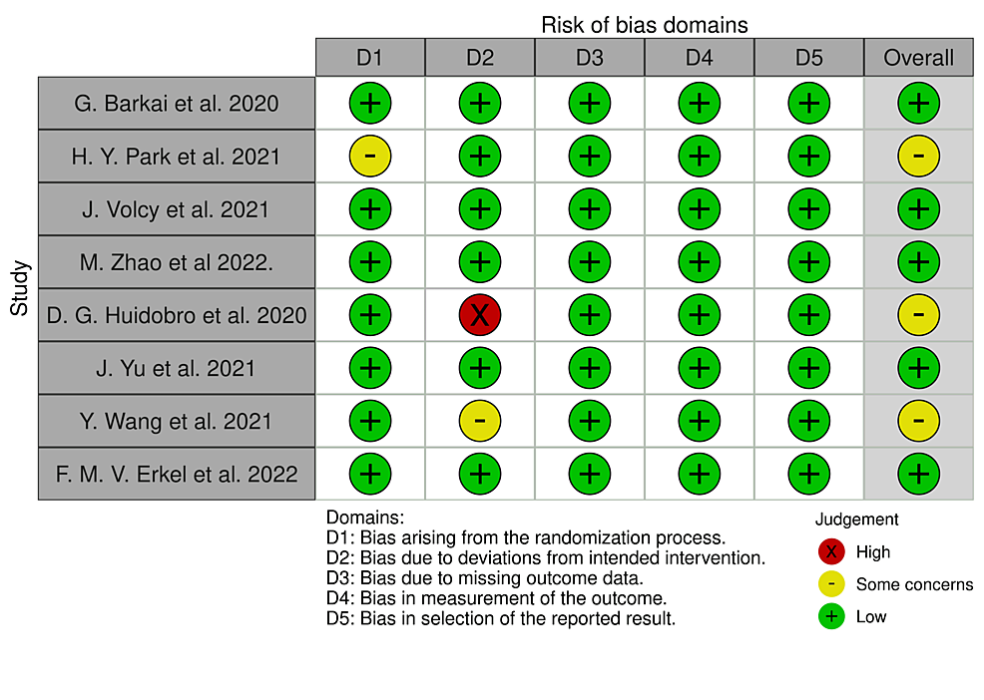
Illustration of the risk-of-bias domains

**Figure 3:**
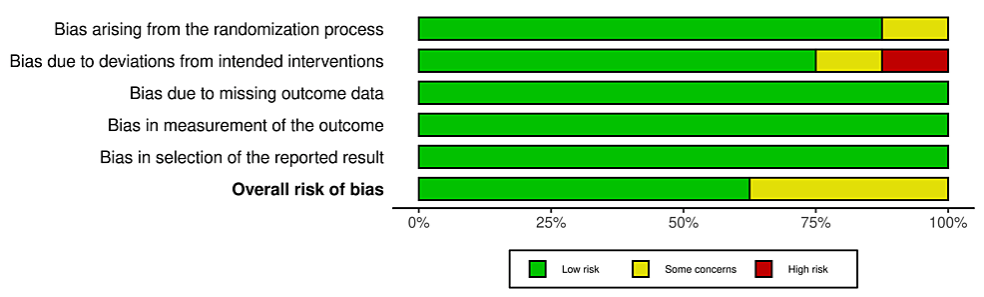
The overall risk of bias from the included studies

The risk-of-publication bias was achieved by using the R-based Robvis software package. Most of the domains showed a low risk of bias. Out of the eight included studies, five studies (62.5%) showed a low risk of bias [12,14,15,17,19]. Only three studies (37.5%) showed some concerns [13,16,18]. The risk of publication bias is represented in Figure 2 and Figure 3.

Discussion

In developed nations, such as the United States (US), a majority of clinical specialties now deliver services through telemedicine. Currently, over 200 telemedicine networks are providing more than 3,500 services. Data from the Centers for Medicare and Medicaid Services indicate a significant surge in weekly telemedicine visits, rising from 13,000 pre-COVID to 1.7 million visits in the week of April 2020. In October 2020, telemedicine visits exhibited a remarkable increase of over 3,000% as compared to 2019, encompassing nearly all medical specialties. The USA's adoption of telemedicine services surpasses that of other countries such as the European Union, Japan, and Korea. Likewise, Japan reports widespread use of digital healthcare facilities, with the Health Ministry noting that 10,000 clinics offer telemedicine services to the Japanese population [20,21].

The increased utilization of telemedicine services is notable in developing nations as well. In South Africa, telemedicine usage faced limitations before the COVID-19 pandemic, primarily due to inadequate compensation. However, since the onset of COVID-19, there has been a substantial upswing in the adoption of telemedicine. Data reveals that tele-triage in South Africa has lightened the load on health facilities, replacing 95% of face-to-face consultations, and in general practice services, teleconsultation facilities successfully address 80% of issues [20].

In India, the widespread adoption of "Telemedicine Practice Guidelines," sanctioned by the Indian Ministry of Health and Family Welfare on March 25, 2020, has resulted in the extensive use of telemedicine facilities. These facilities are accessible through various platforms such as telephones, videoconferences, text messaging, emails, and other telemedicine [22].

Studies that examined patient and provider satisfaction with virtual care during the COVID-19 pandemic reported a median satisfaction score of 4.3 (IQR: 4.0-4.7), indicating a positive view of virtual care. Providers had a median satisfaction score of 4.0 (IQR: 3.3-4.3), slightly lower than patients. Both groups expressed willingness to integrate virtual care into post-pandemic care. Patients desired expanded virtual care services while providers sought clear usage guidelines. The study highlighted the convenience and accessibility of virtual care during the pandemic and its potential ongoing role alongside traditional care. The lack of compensation for healthcare providers conducting virtual consultations was identified as a significant obstacle by both patients and healthcare providers [14].

Based on the results, most individuals found telemedicine to be convenient and expressed satisfaction with its use [13], specifically, those concerned about contracting COVID-19 preferred virtual consultations as opposed to in-person visits. Although many patients had their concerns addressed, a few were dissatisfied due to technical issues during the consultations [18]. Additionally, patients were not content with the payment procedure and web browser used for telemedicine [14].

Patients were concerned about the accuracy of a healthcare professional's medical evaluation over the telephone, and consequently, some were concerned about their health. Patients expressed satisfaction with the time-saving benefits of TCs, such as not having to go to the doctor, not having to spend time in the waiting area, not having to be absent from work, and not having to request family members to join them [19]. Doctors and nurses responded that telemedicine was easy to use because of the simple process and straightforward approach. In contrast to face-to-face sessions, all telemedicine practitioners reported at least one difficulty. Patient-side technical issues attributed for 80% of the work overload; clinician-side technical problems attributed to 26%; delays in VC initiation attributed to 51%; pre-visit preparation contributed 21%; difficulties obtaining out-of-hospital records accounted for 18%; the need for a face-to-face visit attributed for 24%; and difficulty transmitting documents accounted for 20%. It was difficult for 16% of physicians to understand their patients' concerns and undertake a medical evaluation [13]. According to 80% of doctors and nurses, the telemedicine platform is only necessary in emergencies such as COVID-19 [11].

Our findings show that both patients and physicians desire to use telemedicine post-pandemic as an adjunct to, instead of a replacement for, in-person care, and are aware of its limits [15]. Provider reaction was less positive than patient input because clinicians were neutral or unsatisfied with telemedicine, and nearly half of doctors were afraid that telemedicine would compromise the physician-patient interaction [18].

Finally, Baudier et al. (2023) summarized the role of telemedicine as a solution to tackle pandemics, focusing on the COVID-19 pandemic. It highlighted how teleconsultation solutions rapidly implemented during the pandemic changed the way individuals accessed healthcare services and how practitioners provided diagnostics and support to patients. The study emphasized that telemedicine contributes to the resilience of healthcare organizations during pandemics by enabling remote care delivery. Various previous viral outbreaks, such as cholera, SARS, H1N1 influenza, Ebola, Middle East respiratory syndrome (MERS) coronavirus, and the COVID-19 pandemic, have underscored the importance of telemedicine in providing healthcare services during crises. The research also pointed out that telemedicine has been recommended for non-critical patients with chronic diseases, to ensure continuity of care during lockdowns and restrictions. Overall, the study underscores the critical role of telemedicine in healthcare delivery during pandemics and its potential to transform healthcare systems by providing remote access to care [23].

However, acknowledging limitations is crucial for maximizing telemedicine impact. Challenges regarding equitable access, particularly for populations with limited technology or digital literacy skills, require thoughtful solutions. Additionally, ensuring data privacy and cybersecurity remains paramount. Another aspect that needs to be carefully assessed is obtaining verbal informed consent, which should be noted in the patient’s medical records. Furthermore, while studies suggest high overall satisfaction, specific patient groups with lower satisfaction warrant further investigation.

Despite these limitations, the positive trends offer a promising outlook for telemedicine's future. It’s crucial to continually evaluate and refine its implementation to unlock its full potential for revolutionizing healthcare delivery and improving patient outcomes globally. This includes developing strategies for equitable access, strengthening data security, and tailoring implementation to meet the needs of diverse patient populations.

## Conclusions

The COVID-19 pandemic propelled telemedicine into the spotlight, demonstrating its potential as a safe and convenient tool for delivering healthcare. This review suggests widespread acceptance and satisfaction with telemedicine among both patients and providers. Patients appreciate the increased convenience, flexibility, and reduced risk of exposure associated with telemedicine. On the other hand, healthcare providers value their ability to ensure continuity of care and manage patient health remotely. These findings highlight the potential of telemedicine to improve patient experiences and healthcare delivery efficiency.

## References

[1] Sircar K, Popli DB, Jha OK, Sircar M, Hasan S (2022). Oral mucosal lesions in moderate-to-severe COVID-19 disease - an Indian critical care unit experience. J Datta Meghe Inst Med Sci Univ.

[2] Saeed S, Awasthi AA, Nandi D, Kaur K, Hasan S, Janardhanan R (2021). Knowledge, attitude and practice towards COVID-19 among individuals with associated comorbidities. J Med Life.

[3] (2023). WHO coronavirus (COVID-19) dashboard. WHO coronavirus (COVID-19) dashboard with vaccination data. https://data.who.int/dashboards/covid19/cases.

[4] Bhaskar S, Bradley S, Chattu VK (2020). Telemedicine across the globe-position paper from the COVID-19 pandemic health system Resilience Program (REPROGRAM) International Consortium (Part 1). Front Public Health.

[5] Joshi S (2020). COVID-19 and elderly in India: concerns and challenges. Manpower Journal.

[6] Ghosh A, Nundy S, Mallick TK (2020). How India is dealing with COVID-19 pandemic. Sens Int.

[7] Polinski JM, Barker T, Gagliano N, Sussman A, Brennan TA, Shrank WH (2016). Patients’ satisfaction with and preference for telehealth visits. J Gen Intern Med.

[8] Koonin LM, Hoots B, Tsang CA (2020). Trends in the use of telehealth during the emergence of the COVID-19 pandemic - United States, January-March 2020. MMWR Morb Mortal Wkly Rep.

[9] Byrne E, Watkinson S (2021). Patient and clinician satisfaction with video consultations during the COVID-19 pandemic: an opportunity for a new way of working. J Orthod.

[10] (2021). PRISMA transparent reporting of systematic review and meta-analysis. http://www.prismastatement.org/PRISMAStatement/PRISMAStatement.aspx.

[11] McGuinness LA, Higgins JP (2021). Risk-of-bias VISualization (robvis): an R package and Shiny web app for visualizing risk-of-bias assessments. Res Synth Methods.

[12] Barkai G, Gadot M, Amir H, Menashe M, Shvimer-Rothschild L, Zimlichman E (2021). Patient and clinician experience with a rapidly implemented large-scale video consultation program during COVID-19. Int J Qual Health Care.

[13] Park HY, Kwon YM, Jun HR, Jung SE, Kwon SY (2021). Satisfaction survey of patients and medical staff for telephone-based telemedicine during hospital closing due to COVID-19 transmission. Telemed J E Health.

[14] Volcy J, Smith W, Mills K (2021). Assessment of patient and provider satisfaction with the change to telehealth from in-person visits at an academic safety net institution during the COVID-19 pandemic. J Am Board Fam Med.

[15] Zhao M, Elshoni H, O'Brien J (2022). Patient and provider experiences with virtual care during the COVID-19 pandemic: a mixed methods study. Patient Exp J.

[16] Garcia-Huidobro D, Rivera S, Valderrama Chang S, Bravo P, Capurro D (2020). System-wide accelerated implementation of telemedicine in response to COVID-19: mixed methods evaluation. J Med Internet Res.

[17] Yu J, Afridi SM, Cozart AC, Isea L, Guan J (2021). Evaluation and feedback for telehealth from patients and physicians during the early stage of COVID-19 pandemic period. Cureus.

[18] Wang Y, Yang J, Ma H, Dong X, Xie G, Ye S, Du J (2021). Application of telemedicine in the COVID-19 epidemic: an analysis of Gansu Province in China. PLoS One.

[19] van Erkel FM, Pet MJ, Bossink EH (2022). Experiences of patients and health care professionals on the quality of telephone follow-up care during the COVID-19 pandemic: a large qualitative study in a multidisciplinary academic setting. BMJ Open.

[20] Omboni S, Padwal RS, Alessa T (2022). The worldwide impact of telemedicine during COVID-19: current evidence and recommendations for the future. Connect Health.

[21] Kichloo A, Albosta M, Dettloff K (2020). Telemedicine, the current COVID-19 pandemic and the future: a narrative review and perspectives moving forward in the USA. Fam Med Community Health.

[22] Kaur KN, Niazi F, Thakur R, Saeed S, Rana S, Singh H (2022). Patient satisfaction for telemedicine health services in the era of COVID-19 pandemic: a systematic review. Front Public Health.

[23] Baudier P, Kondrateva G, Ammi C, Chang V, Schiavone F (2022). Digital transformation of healthcare during the COVID-19 pandemic: patients’ teleconsultation acceptance and trusting beliefs. Technovation.

